#  Intracellular staphylococcus aureus: Live-in and let die

**DOI:** 10.3389/fcimb.2012.00043

**Published:** 2012-04-24

**Authors:** Martin Fraunholz, Bhanu Sinha

**Affiliations:** ^1^Department of Microbiology, Biocenter, University of Würzburg,Würzburg, Germany; ^2^Department of Medical Microbiology, University Medical Center Groningen,Groningen, Netherlands

**Keywords:** Staphylococcus aureus, phagocytosis, phagosomal escape, autophagy, host cell death, bacterial persistence

## Abstract

*Staphylococcus aureus* uses a plethora of virulence factors to accommodate a diversity of niches in its human host. Aside from the classical manifestations of *S. aureus*-induced diseases, the pathogen also invades and survives within mammalian host cells.The survival strategies of the pathogen are as diverse as strains or host cell types used. *S. aureus* is able to replicate in the phagosome or freely in the cytoplasm of its host cells. It escapes the phagosome of professional and non-professional phagocytes, subverts autophagy, induces cell death mechanisms such as apoptosis and pyronecrosis, and even can induce anti-apoptotic programs in phagocytes. The focus of this review is to present a guide to recent research outlining the variety of intracellular fates of *S. aureus.*

## INTRODUCTION

Most manifestations of *Staphylococcus aureus* disease involve extracellular bacteria (furuncles, carbuncles, impetigo, abscesses, septicemia, necrotizing pneumonia) or biofilm formation (catheter-induced infective endocarditis, atherosclerosis). Aside from this *S. aureus* infections have a second face: there is accumulating evidence that *S. aureus* is able to survive within its hosts cells and thus might be termed a facultative intracellular pathogen. Intracellularity of *S. aureus* has been implied as immune-evasive strategy thereby escaping detection by professional phagocytes.

## INTERNALIZATION OF *S. aureus* BY HOST CELLS

**FIGURE 1:**
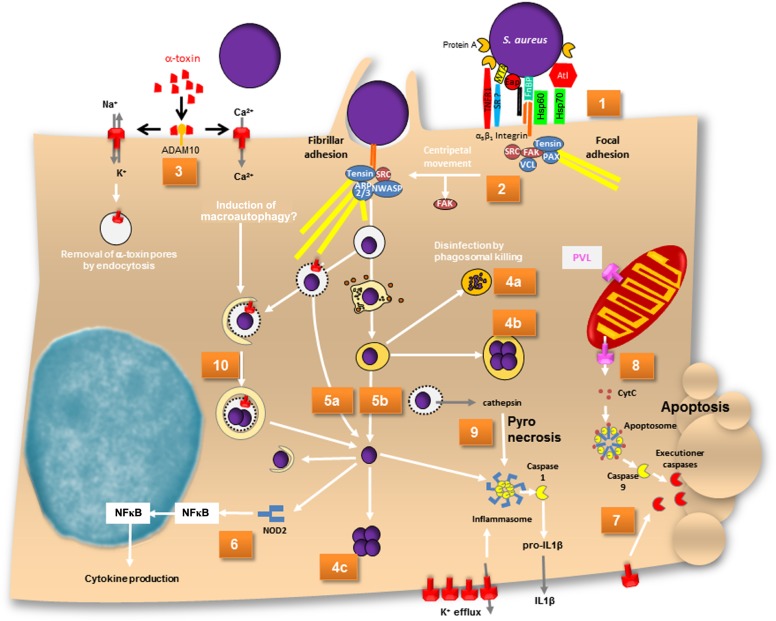
**A map of intracellular fates of *S. aureus.*** (1) α_5_β_1_ integrins are sequestered by FnBP-dependent fibronectin cross-linking at focal adhesions. (2) Centripetal movement and loss of FAK lead to development of fibrillar adhesions, at which phagocytic cups are formed and bacteria are eventually endocytosed. (3) Assembly of α-toxin pores on the plasma membrane of host cells leads is dependent on ADAM10. α-Toxin pores are permeable for cations. Ca^2+^ has been reported to induce macroautophagy. (4a) Bacteria are disinfected by phagolysosomes or (4b) survive and grow within endosomes or (4c) in the cytoplasm after phagosomal escape. (5a) Phagosomal escape can be mediated by α-toxin in cystic fibrosis cells and (5b) also by a combination of phenol-soluble modulins and phospholipases. (6) Cytoplasmic *S. aureus* peptidoglycan is recognized by NOD2, which activates NFκB and results in cytokine production. (7) The mode of cell death induced by *S. aureus* is not completely understood. While caspase-independent cell death exists, α-toxin is capable of inducing extrinsic apoptosis. Upon alpha-toxin induced potassium efflux caspase 2 has recently been shown to lead to mitochondrial outer membrane permeabilization. (8) PVL has been reported to permeabilize mitochondrial outer membrane thereby releasing cytochrome *c* and thus inducing the apoptosome in a Bax-independent pathway of intrinsic apoptosis. Caspase 9 subsequently activates executioner caspases. (9) Cathepsin release from permeabilized phagosomes activates the inflammasome. Activated caspase 1 leads to IL1β maturation and inflammatory pyronecrotic cell death. (10) Toxin-permeabilized endocytic vesicles are targeted by autophagy. During autophagy an isolation membrane engulfs leaky endosomes or cytoplasmically located bacteria. Within these autophagosomes bacterial replicate and eventually escape the organelle ultimately leading to host cell death. ADAM, a metalloprotease and disintegrin; ARP2/3, actin-related protein 2 and 3; Atl, autolysin; CytC, cytochrome *c*; Eap, extracellular adherence protein; FAK, focal adhesion kinase; FnBP, fibronectin-binding protein; HSP, heat shock protein; IL, interleukin; NF?B, nuclear factor κB; NWASP, neural Wiskott–Aldrich syndrome protein; PAX, paxillin; SR, scavenger receptor; VCL, vinculin; WTA, wall teichoic acid.

Invasion of non-professional phagocytes by *S. aureus* is mediated by a zipper-type mechanism. To date many bacterial adhesins have been identified with Fibronectin (Fn)-binding proteins A and B (FnBPA, FnBPB) constituting the major staphylococcal adhesins for non-professional phagocytes such as epithelial, endothelial cells, fibroblasts, osteoblasts, and keratinocytes (Dziewanowska et al., 1999; Jevon et al., 1999; Lammers et al., 1999; Peacock et al., 1999; Sinha et al., 1999; Fowler et al., 2000; Ahmed et al., 2001; Kintarak et al., 2004; Sinha and Fraunholz, 2010; Edwards et al., 2011; **Figure 1**, Map Item 1). Fibronectin-bridging between FnBPs and α_5_β_1_ integrins on the host cell surface is sufficient to induce zipper-type uptake of staphylococci (Sinha et al., 2000). However, FnBPs also have been shown to directly bind to human heat shock protein 60 (Hsp60) exposed on the cellular surface (Dziewanowska et al., 2000). FnBP-independent invasion was observed in *S. aureus* Newman, which produces C-terminally truncated FnBPs that are not covalently anchored to the cell wall of *S. aureus*. It has been shown that strain Newman gets internalized by epithelial cells and fibroblasts mediated by extracellular adherence protein (Eap; Harraghy et al., 2003) with its cellular receptor still not identified to date. Recently, the staphylococcal autolysin (Atl) was identified to function as adhesin/invasin with heat shock cognate protein Hsc70 being the direct cellular receptor (Hirschhausen et al., 2010). Further, wall teichoic acids (WTA) seem to be important for establishment of nasal colonization and there is evidence that a scavenger receptor is involved in WTA binding (Weidenmaier et al., 2004, 2005, 2008). Clumping factor B (ClfB) has been shown to bind to cytokeratins in the extracellular matrix (ECM) of host cells (O’Brien et al., 2002; Wertheim et al., 2008; Haim et al., 2010), and staphylococcal protein A can directly interact with tumor necrosis factor α receptor 1 (TNFR1; Claro et al., 2011). To what extent the internalization of the pathogen is mediated by WTA, ClfB, protein A, and a body of other molecules interacting with the ECM of host cells is not known thus far.

Since FnBPs contribute to the adherence of *S. aureus* to intact endothelium *in vivo* (Laschke et al., 2005; Kerdudou et al., 2006; Edwards et al., 2010), we can assume that staphylococcal invasion of epithelia or the endothelium is relevant in natural infections. The interaction of FnBP with ECM Fn is mediated by tandem β zipper structures via the binding of multiple fibronectin molecules by the repetitively arranged modules within a single FnBP (Schwarz-Linek et al., 2003; Rudino-Pinera et al., 2004; Bingham et al., 2008). As a result FnBP/Fn sequester α_5_β_1_ integrins on the host cell surface. The resulting receptor clustering relays signals that result in cytoskeletal rearrangements (Agerer et al., 2005; Schröder et al., 2006b). The rearrangements initiated at focal adhesions, which are remodeled to fibrillar adhesions by loss of focal adhesion kinase (FAK), paxillin, and vinculin. The rearrangement is accompanied by a centripetal movement of *S. aureus* on the host cell surface that were observed by videomicroscopy (Schröder et al., 2006a). The repeated generation of actin comet tails beneath adherent staphylococci or FnbA-coated beads and the formation of actin cups without internalization of staphylococci is interpreted by the authors as a delay of phagocytosis (Schröder et al., 2006a; **Figure 1**, Map Item 2). Invasion signaling further involves src kinase (Agerer et al., 2003). Extracellular signal-regulated kinases (ERK) and c-Jun N-terminal kinase (JNK) but not mitogen-activated protein kinase (MAPK) p38 are required in osteoblasts (Ellington et al., 2001), whereas in HEp-2 cells p38 MAPK was found to be upregulated alongside ERK (Li et al., 2009). Further, phosphorylation of transcription factor c-Jun, but not of Elk-1 or ATF-2 has been demonstrated during invasion of osteoblasts (Ellington et al., 2001). The phosphoinositide-3-kinase (PI3K)-Akt pathway is active during *S. aureus* internalization by bovine endothelial cells (Oviedo-Boyso et al., 2011).

Eventually, *S. aureus* gets endocytosed by professional as well as non-professional cells where the pathogen faces a variety of intracellular fates.

## *Staphylococcus aureus* INTRACELLULAR PERSISTENCE AND GROWTH

The fates of the pathogen and the infected host cell depend on staphylococcal isolate and genotype (Krut et al., 2003) as well as differential susceptibility of host cells to virulence factors, host cell gene expression, etc. For example, *S. aureus* produces different hemolysins. The majority of bovine mastitis strains were phenotypically positive for the sphingomyelinase β-toxin, whereas only a minority of human strains isolated from cases of septicemia or nasal carriage was positive for β-toxin (Aarestrup et al., 1999). There seems to exist a selective pressure for *S. aureus* strains colonizing humans to acquire β-toxin converting phage (Goerke et al., 2009). This is most likely due to staphylococcal complement inhibitor SCIn and the chemotaxis inhibitor protein CHIPS (and additional factors) that are usually found to be encoded by the respective phage genomes. Purified β-toxin, selectively kills monocytes (Bhakdi et al., 1996) and destroys platelets, but barely affects other cell types (Wadstrom and Mollby, 1972). Similarly, most human cell types are fairly insensitive to the pore-former α-toxin, whereas human leukocytes and cells from other mammalian species are highly susceptible (Bhakdi and Tranum-Jensen, 1991). The metalloprotease ADAM10 has been recently identified as receptor of α-toxin monomers (Wilke and Bubeck Wardenburg, 2010; Inoshima et al., 2011; **Figure 1**, Map Item 3). The differential specificities of α-toxin possibly reflect ADAM10 expression differences in the respective cells or might result from differential capability of host cells to remove α-toxin pores by endocytosis (Husmann et al., 2009).

*Staphylococcus aureus* survival within host cells was highly dependent on multiplicity of infection (MOI; e.g., Mohammed et al., 2007; Schwartz et al., 2009; Pang et al., 2010) and also the growth phase of the bacteria used for infection (Schwartz et al., 2009). Green-fluorescent protein (GFP)-expressing *S. aureus* displayed bleaching of the fluorescent protein, which indicated degradation of the bacteria in polymorphonuclear neutrophils (PMN; **Figure 1**, Map Item 4a). The loss of fluorescence was not strain-specific and was seen in each of several different strains of *S. aureus,* including nosocomial and community-associated methicillin-resistant strains. When rapidly growing *S. aureus* was used for infection, the bacteria were found to be more susceptible to GFP bleaching (Schwartz et al., 2009). This indicated that these bacteria were cleared more efficiently. Bacterial disinfection was mainly dependent on hypochlorous acid (HOCl; Schwartz et al., 2009). Phagosomal acidification and digestion of *S. aureus* within professional phagocytes is required for MyD88-dependent toll-like receptor (TLR) responses to infection (Abdelzaher et al., 2010).

Not all bacterial cells are disinfected by the phagolysosomes. *S. aureus* has been reported to persist inside phagocytes or endothelial cells for prolonged periods (Hamill et al., 1986; Lowy et al., 1988; Vann and Proctor, 1988; Buisman et al., 1991; Hiemstra et al., 1992; Schröder et al., 2006a; Garzoni et al., 2007; Kubica et al., 2008; Tuchscherr et al., 2011; reviewed in Sendi and Proctor, 2009). Persistence is most often attributed to small colony variants (SCVs) of *S. aureus.* SCVs often present a metabolically quiescent, non-hemolytic, non-pigmented phenotype characterized by reversible auxotrophies in heme biosynthetic pathways or in oxidative phosphorylation (Proctor et al., 1994) as well as a defined transcriptome (Garzoni et al., 2007) and proteome (Kriegeskorte et al., 2011). Also, SCVs are generally found to be mutants in the accessory gene regulator locus *(agr),* thus failing to produce a variety of quorum sensing-controlled virulence factors. SCVs grow slowly and hence are more resistant to a variety of antibiotics (reviewed in Sendi and Proctor, 2009). Further, SCVs display a thick cell wall (Bulger and Bulger, 1967) and an up-regulation of alternative sigma-factor σB (Moisan et al., 2006), which enables *S. aureus* to cope with a variety of environmental stressors (Horsburgh et al., 2002). There is increasing evidence that *S. aureus* can persist *in vivo* in human infections presumably owing to its extreme durability and resistance against a variety of environmental conditions and thus can serve as a potential source for recurrent infection (Proctor et al., 1995; von Eiff et al., 2001; Kipp et al., 2003; Schröder et al., 2006a; reviewed in Garzoni and Kelley, 2009; Sendi and Proctor, 2009). SCVs of *S. aureus* even have been shown to survive and grow within host cell phagosomes (Schröder et al., 2006a; **Figure 1**, Map Item 4b). Also, the complementation of *rsb*U in laboratory strains restored activity of the alternative sigma-factor σB and led to intracellular growth of *S. aureus* within phagolysosomes of THP-1 phagocytes (Olivier et al., 2009). Contrasting these reports, staphylococcal growth has been described after pathogen translo-cation to the host cell cytoplasm (**Figure 1**, Map Item 4c): *S. aureus* strain Newman is able to escape the phagosome and persists within human monocyte-derived macrophages (hMDM) which resulted in host cell lysis on day 5 after infection (Kubica et al., 2008). The authors postulate that this survival within phagocytes might constitute a route for dissemination of staphylococcal infection. This is further corroborated by the identification of cytopro-tective effects on macrophages after phagocytosis of *S. aureus*. Thus, the up-regulation of anti-apoptotic factors upon staphy-lococcal infection is responsible for extended phagocyte lifetime (Koziel et al., 2009). Both studies suggest that *S. aureus* might penetrate deeper into the tissue and even disseminate to different sites within “Trojan horse” phagocytes (Koziel et al., 2009). Survival within PMN is reported to depend on the accessory regulator Sar1, which was crucial to *S. aureus* survival inside spacious vacuoles, whereas *sar*^-^ strains were localizing to so-called “tight vacuoles” (Gresham et al., 2000). Such different vacuoles can also be observed innon-professional phagocytes (Sinha and Fraunholz, 2010). The large vacuoles also are reminiscent of spacious *Liste-ria*-containing phagosomes, which were found to be non-acidified and non-degradative niches in macrophages (Birmingham et al., 2008). However, a more thorough characterization of the different vacuolar locations in intracellular *S. aureus* infections is lacking.

## *Staphylococcus aureus* PHAGOSOMAL ESCAPE

**FIGURE 2:**
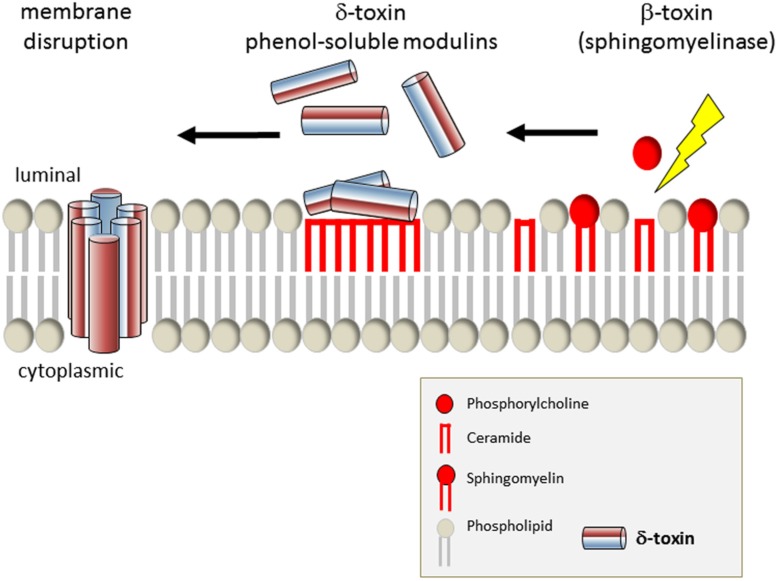
**Model of phagosomal escape by *S.* aureus by synergistic action of δ-toxin and β-toxin.** After cleavage of sphingomyelin to ceramide and phosphocholine, δ-toxin is capable of interacting more efficiently with the outer leaflet of the eukaryotic plasma membrane. δ-Toxin accumulates in the resulting hydrophobic ceramide membrane domains and eventually permeabilizes the target membrane.

Phagocytosed bacterial pathogens evade lysosomal killing, e.g., by disintegration of the organelle membrane in order to translocate into the host cell cytoplasm. *Listeria monocytogenes* co-opts the pore-forming toxin (PFT) listeriolysin O (LLO) and phos-pholipases (reviewed in Dramsi and Cossart, 2002; Schnupf and Portnoy, 2007), and Group A streptococci use the PFT strep-tolysin O (Hakansson et al., 2005). Phagosomal escape of *S. aureus* initially has been described by Bayles et al. (1998) and the *agr*-dependency of this process has been demonstrated (Qazi et al., 2001; Shompole et al., 2003; Jarry and Cheung, 2006; Kubica et al., 2008). The membrane destructive function of staphylococcal a-toxin thus suggested an involvement of the pore-former in phagosomal membrane disruption. The requirement for phago-somal escape and intracellular bacterial survival has been reported in CFT-1, a cystic fibrosis (CF) lung cell line (Jarry and Cheung, 2006) as well as in macrophages (Kubica et al., 2008; **Figure 1**, Map Item 5a). In CFT-1, *S. aureus* translocates into the cytoplasm in an α-toxin-dependent manner and the bacteria replicate within the cytoplasm (Kahl et al., 2000; Jarry et al., 2008). However, in the LCSFN cell line complemented with the wild-type CF transmem-brane conductance regulator, CFTR, α-toxin has no effect (Jarry and Cheung, 2006). Further, it has been demonstrated that neither α-toxin overexpressing strains (Lám et al., 2010) nor laboratory strains inducibly expressing α-toxin (Giese et al., 2009, 2011) are capable of releasing staphylococci into the host cell cytoplasm. By expression of the amphiphilic 26 amino-acid (AA) peptide δ-toxin in the non-cytotoxic laboratory strain *S. aureus* RN4220 *S. aureus* was capable of escape in rates similar to that of heterolo-gously expressed LLO (Giese et al., 2011). δ-Toxin is encoded by the agr-effector RNAIII and is translated about 1 h after transcription of RNAIII (Balaban and Novick, 1995). It is capable of lysing bacterial protoplasts, lysosomes, lipid spherules, mitochondria, and erythrocytes *in vitro* in a temperature-independent manner. Its activity and mode of action is comparable to that of non-ionic detergents (Kreger and Bernheimer, 1971; Kreger et al., 1971; Rahal Jr., 1972; Kapral, 1976; reviewed in Verdon et al., 2009). δ-Toxin is encoded by RNAIII, the *agr* effector, and thus might constitute an immediate response to space limitation by phagosomal engulfment (**Figure 1**, Map Item 5b). However, membrane disruption by δ-toxin depended on the presence of the staphylococcal sphingomyelinase, β-toxin (Giese et al., 2011), which cleaves sph-ingomyelins (SM) to phosphorylcholine and ceramide moieties. δ-Toxin hardly binds to negatively charged phospholipids, binds strongly to liquid-disordered domains and poorly to cholesterol and sphingomyelin liquid-ordered raft domains (Pokorny et al., 2006). In one model, β-toxin thus may cleave SM to ceramides, which tend to accumulate in membrane microdomains. The hydrophobic nature of ceramide-rich domains thus might constitute regions of δ-toxin assembly, which eventually lead to target membrane permeabilization (**Figure 2**). *S. aureus* strain USA300 LAC, however, is escape proficient yet does not encode a functional β-toxin due to lysogeny of a β-converting phage (Diep et al., 2006). We thus have to hypothesize alternative factors that can act in phagosomal escape, such as a variety of lipases encoded by the staphylococcal genome or phenol-soluble modulins (PSMs; see below). Alternatively, prophages might be lost, e.g., during exposure to phagosomal reactive oxygen species, and then might contribute to phagosomal escape. A similar activation mechanism is used by *Streptococcus pneumoniae,* which produces hydrogen peroxide and thereby lyses *S. aureus* by a “remote control” prophage activation (Selva et al., 2009).

For complete hemolysis of erythrocytes in sheep blood agar plates either a shift to 4°C (hot-cold hemolysis), osmotic stress, or synergistic toxins are necessary. δ-Toxin belong to the class of PSMs, which initially had been shown to be present within a hot-phenol extraction of *S. epidermidis* extracts (Otto et al., 2004) and only later had been identified in *S. aureus* by sequence homology (Wang et al., 2007). The major groups of PSMs are divided into two operons referred to as PSMα and PSMβ. The PSMα operon is comprised of four open reading frames (ORFs) with approximately 20 AA. PSMβ encodes two ORFs, which are about 40 AA in length (Wang et al., 2007). The expression of PSMβ has been demonstrated to result in phagosomal escape in a gain-of-function study (Giese et al., 2011). Just like δ-toxin, PSMα and PSMβ are *agr*-dependently expressed. It recently was shown that the staphylococcal *agr* system is confinement induced and thus comprises not only a quorum sensing system but also a diffusion sensing system active at the single cell level (Carnes et al., 2009). δ-Toxin and PSMs are hence produced upon phagosomal confinement and thus also low numbers of endocytosed staphy-lococci should be able to mount a toxin response in order to avoid lysosomal killing. Whereas gain of function assays demonstrated PSMβ activity in phagosomal escape (Giese et al., 2011), *in vivo* studies suggests a prominent role of PSMα, with the third ORF of the operon, PSMα3, being the most virulent principle (Wang et al., 2007). Despite these incongruities a common theme emerges: amphiphilic PSMs are involved in phagosomal escape. One possibility to explain the observed differences is the postu-lation of alternative pathways of phagosomal escape mechanisms for *S. aureus.*

Molecular patterns of pathogens that reside in the cytoplasm of their hosts are detected by nucleotide-binding and oligomerization domain proteins NOD1 and NOD2, which detect γ-D-glutamyl-diaminopimelic acid and muramyl dipeptide, pep-tidoglycan components of Gram-negative and Gram-positive bacteria. Peptidoglycan binding leads to a conformation change in NOD proteins, which initiates the recruitment of ubiquitin lig-ases and kinases and ultimately results in nuclear translocation of NFκB and activator protein 1 and expression of inflammatory genes (reviewed in Strober et al., 2006). NOD2 signaling upon *S. aureus* infection has been found to induce cytokine production (Kapetanovic et al., 2007) and thus might contribute to induction of inflammation, e.g., in the lung (Gomez and Prince, 2008; **Figure 1**; Map Item 6). NOD2-deficient mice exhibit a delayed inflammatory response and impaired bacterial clearance after infection with *S. aureus* (Hruz et al., 2009). α-Toxin facilitates NOD2-dependent recognition of *S. aureus* muramyl dipeptide (Hruz et al., 2009), possibly by interfering with phagosomal integrity.

The observation that *S. aureus* can translocate into the cytoplasm of host cells and grow without an immediately ensuing cell death (e.g., Kubica et al., 2008) illustrates that phagosomal escape is not identical with cytotoxicity Thus, the link between phagosomal escape and host cell death still needs to be elucidated.

## *Staphylococcus aureus*-INDUCED HOST CELL DEATH

The classical separation of host cell death into programed cell death (PCD; apoptosis) and accidental cell death or necrosis has long been superseded after the identifying a large variety of cell death mechanisms (for a reviews, see Taylor et al., 2008; Ting et al., 2008a; Bergsbaken et al., 2009). Many of which are defined by key factors that allow the assessment of death pathway activation following bacterial infection (e.g., reviewed in Rudel et al., 2010), although the synchronous activity of virulence factors from the staphylococcal arsenal renders unequivocal correlations between toxin and associated mode of cell death a daunting task. When virulent *S. aureus* strains are added to host cells in tissue culture, host cell death occurs via mechanisms that have been mainly identified as apoptotic (Bayles et al., 1998; Menzies and Kourteva, 1998, 2000; Wesson et al., 1998, 2000; Kahl et al., 2000; Nuzzo et al., 2000; Tucker et al., 2000; Haslinger et al., 2003; Genestier et al., 2005; **Figure 1**, Map Item 7). α-Toxin is both required and sufficient for induction of leukocyte cell death, either apoptotic or necrotic (Bantel et al., 2001; Essmann et al., 2003; Haslinger et al., 2003). Leukocytes are sensitive to staphylococcal α-toxin. Already low toxin doses induce apoptosis accompanied by a breakdown of the mitochondrial transmembrane potential (Bantel et al., 2001; Haslinger et al., 2003). A recent study suggests that caspase-2 acts as an initiator caspase during cell death of non-professional phagocytes. Caspase 2 was induced by potassium efflux due to pore-forming toxins such as staphylococcal alpha-toxin and aerolysin (Imre et al., 2012). By contrast, high doses induce necrotic cell death (Walev et al., 1993; Bantel et al., 2001; Essmann et al., 2003; Haslinger et al., 2003). Human endothelial cells are virtually insensitive to the action of *S. aureus* α-toxin, however, comparatively low numbers of *S. aureus* cells with a combined invasive and strongly hemolytic phenotype readily induce apoptotic cell death in HUVEC (Haslinger-Löffler et al., 2005). This suggests that cell death mechanisms are activated from within their intracellular location. The effect is highly specific, since fixed, non-hemolytic, rifampin-treated or weakly invasive staphylococci are not cytotoxic toward endothelial cells (Haslinger-Löffler et al., 2005). Multiple other studies indicate that *S. aureus* might kill its other types of host cells from within (Bayles et al., 1998; Menzies and Kourteva, 1998; Wesson et al., 1998; Nuzzo et al., 2000; Tucker et al., 2000; Krut et al., 2003; Haslinger-Löffler et al., 2005; Chatterjee et al., 2008; Jarry et al., 2008; Kubica et al., 2008; Lám et al., 2010). The virulence factors required for *S. aureus*-induced apoptosis in endothelial cells depend on *agr* and the alternative stress–response sigma-factor σB (Wesson et al., 1998; Qazi et al., 2001; Shompole et al., 2003; Jarry and Cheung, 2006; Kubica et al., 2008), but mainly seem to be independent of SarA (Haslinger-Löffler et al., 2005; Jarry et al., 2008) - although there are contradicting results on the involvement of SarA (Wesson et al., 1998).

Phage-encoded Panton-Valentine leukocidin (PVL) predominantly destroys leukocytes, although there also is some species specificity for human and rabbit PMN (Loffler et al., 2010). In PMN, PVL induced a rapid caspase-9/3-dependent cell death *in vitro* (Genestier et al., 2005). The authors further identified to a mitochondrial localization of the PVL toxin. Isolated mitochondria were permeabilized for pro-apoptotic factors such as cytochrome *c* (CytC) by PVL which suggested that PVL is able to create pores in the mitochondrial outer membrane and thus triggers a Bax-independent mitochondrial pathway of host cell apoptosis (Genestier et al., 2005; **Figure 1**, Map Item 8). During the intrinsic pathway of apoptosis release of CytC from injured mitochondria leads to activation of apoptosis-activating factor-1 (APAF-1). Oligomerizing CytC/APAF-1 recruits and subsequently activates pro-caspase 9. Caspase 9 then proteolytically activates effector caspases, which finally cleave their respective substrates resulting in membrane blebbing, and DNA fragmentation (reviewed in Rudel et al., 2010). Similarly, α-toxin has been described to activate caspases via the intrinsic death pathway (Bantel et al., 2001; Haslinger et al., 2003) independently of death receptor signaling (CD95/Fas/APO-1). Bcl-2 overexpressing Jurkat cells were protected from α-toxin mediated cell death (Bantel et al., 2001; Essmann et al., 2003) although recent result suggest that the function of Bcl-2 in autophagy might be responsible for the observed phenomena (Schnaith et al., 2007; Mestre et al., 2010).

Interestingly, *S. aureus* also seems to be able to exert anti-apoptotic host cell responses. The pathogen was shown to suppress staurosporine-induced apoptosis in hMDM although early apoptotic features such as phosphatidylserine display at the outer plasma membrane leaflet, reduced mitochondrial membrane potential, CytC release, and caspase-3 activation are still observed (Koziel et al., 2009). *S. aureus* infection strongly upregulated the expression of mitochondrial membrane potential stabilizing Bcl-2 and Mcl-1 gene products. Since also heat-killed *S. aureus* was able to suppress apoptosis in the host cells staphylococcal products such as lipoteichoic acid or peptidoglycan might activate the macrophages through intracellular pattern recognition sensors such as nucleotide oligomerization domain receptors (NOD; Kapetanovic et al., 2007; Koziel et al., 2009).

Aside from apoptosis *S. aureus* is also able to induce pyronecrosis (**Figure 1**; Map Item 9). There, caspase-1 is activated as part of an inflammasome, which further consists of NOD-like receptor protein 3 (NLRP3) and the adaptor protein, apoptosis-associated speck-like protein containing a caspase-associated recruitment domain (ASC). *S. aureus* can function as a stimulus for NLRP3 (Munoz-Planillo et al., 2009), however, the molecular identity of the stimulating signal is not known thus far (Mariathasan et al., 2006; Ting et al., 2008b; Wright and Nair, 2010). Lysosomal permeabilization is one NLRP3-activating principle, wherein release of the lysosomal protease cathepsin B into the cytoplasm contributes to NLRP3 activation (reviewed in Willingham and Ting, 2008; Bergsbaken et al., 2009). Thus, it seems likely, that lysosomal rupture or permeabilization by *S. aureus* toxins releases cathepsin which then activates the inflammasome. Indeed staphylococcal α, β, and γ-hemolysins have been shown to be important activation of the NLRP3 inflammasome (Craven et al., 2009; Munoz-Planillo et al., 2009; Kebaier et al., 2012). The pore-forming α-and γ-toxins of *S. aureus* permeabilize membranes and thus might be involved in cathepsin release and subsequent inflammasome activation. α-Toxin further is known to permeabilize the plasma membrane for potassium ions. In turn, potassium efflux activates the inflammasome (Petrilli et al., 2007). β-Toxin has been shown to be involved in phagosomal escape (Giese et al., 2011) and thus also might act in cathepsin release.

## *Staphylococcus aureus* AND THE SUBVERSION OF AUTOPHAGY

Autophagy sequesters cytoplasmic contents via an isolation membrane. Engulfment of cargo by the so-called phagophore forms double membrane-bound autophagic vesicles that eventually fuse with lysosomes to yield autolysosomes. Autophagy serves for degradation of organelles or self-digestion during nutrient limiting conditions such as starvation and is generally thought to constitute a cellular survival mechanism. During bacterial infections autophagy disposes of leaky vesicles or intracellular bacteria, however, bacterial pathogens have found multiple ways to subvert autophagy (reviewed in Dorn et al., 2002; Kirkegaard et al., 2004; Levine, 2005; Campoy and Colombo, 2009; Orvedahl and Levine, 2009; Ogawa et al., 2011).

*Staphylococcus aureus* was shown to interact with autophagosomes in a rather unique way (Schnaith et al., 2007) when compared to autophagy-subvertive strategies of other bacterial pathogens (Campoy and Colombo, 2009; Ogawa et al., 2011). *S. aureus* inhibits fusion of phagosomes with lysosomes. It permeabilizes HeLa phagosomes by a mechanism dependent on *Staphylococcus*-secreted toxins. The leaky phagosomes are targeted by autophagy and within autophagosomes *S. aureus* replicates. Eventually the bacteria escape from their intracellular confinement into the host cell cytoplasm in an *agr*-dependent manner (Schnaith et al., 2007; **Figure 1**, Map Item 9). Finally, host cell death is induced, which is independent of a caspase activation cascade but was blocked by overexpression of anti-autophagic Bcl-2. Induction of autophagy by rapamycin resulted in an increased number of recovered colony-forming units, whereas inhibition with wortmann in reduced the colonies recovered from the intracellular environment. *S. aureus*-induced autophagy resulted in a vacuolization of the host cell cytoplasm (“Swiss cheese phenotype”). *agr*-deficient *S. aureus* fail to induce autophagy, which results in maturation of bacteria-containing phagosomes followed by lysosomal degradation of the pathogens. α-Toxin is able to permeabilize membranes for Ca^2+^, an inducer of autophagy (Brady et al., 2007) and autophagy targets phagosomes perforated by α-toxin in Chinese hamster ovary cells (Mestre et al., 2010). Whereas latter observation is in line with the results obtained by Schnaith et al. (2007) it contrasts findings that α-toxin is not sufficient to permeabilize HeLa phagosomes (Jarry and Cheung, 2006; Giese et al., 2009; Lám et al., 2010).

## CONCLUSION

With about 200–300 virulence factors, *S. aureus* is able to exert a multitude of effects upon its eukaryotic host cells. Although many details have emerged through ground-breaking and recent research, only a minority of pathogenicity factors of *S. aureus* has been functionally annotated to date. Particularly the assessment of intracellular staphylococcal virulence is often hampered by the difficulty to discriminate between toxin effects that result from extracellular or intracellular bacteria, although inducible toxin-expressing might provide valuable tools for molecular dissection of host–pathogen interactions.

With our advancing knowledge of cell death mechanisms ground-breaking experiments will have to be re-evaluated in order to understand the mechanisms of *S. aureus*-induced host cell killing. When comparing experimental research originating from different labs the influence of a body of factors should be taken into account that could lead to different infection outcomes:

*Staphylococcus aureus* strain used for infection, its growth phase at the time of infection (and hence the bacterial growth medium), as well as MOI are important, whereas on the host side the cell type and hence the protein profile will drastically influence infection outcome by altering host cell susceptibility to bacterial toxins, response to pathogen-associated molecular patterns, expression of receptors, caspases, and other host factors.

In order to identify activities of single toxins or virulence factors gain-of-function studies can be useful, e.g., using the a pathogenic *S. carnosus* as toxin delivery vehicle. However, such strategies will not easily identify pathways during which an orchestrated interplay of multiple virulence factors is required. However, novel high-throughput sequencing technologies of transposon insertions (Gawronski et al., 2009; van Opijnen et al., 2009) open up new possibilities for analysis of genome-wide mutant libraries of clinically relevant strains. Using such novel tools we will be able to address a lot of open questions with regard to intracellular staphylococcal infections: do different adhesins result in employment of different uptake mechanisms into non-professional phagocytes as these would subsequently result in different infection outcomes? What is the nature of tight and spacious vacuoles (Gresham et al., 2000) that are occupied by, for example, *sarA*- and *sar*A+ *S. aureus*, respectively? Which alternative pathways for phagosomal escape do exist? Which modes of cell death are activated by a single strain in different host cell types or different strains in a single host cell line? There is still a lot to learn about the versatile facultative intracellular pathogen, *Staphylococcus aureus*.

## Conflict of Interest Statement

The authors declare that the research was conducted in the absence of any commercial or financial relationships that could be construed as a potential conflict of interest.

## References

[B1] Aarestrup F. M. Larsen H. D. Eriksen N. H. Elsberg C. S. Jensen N. E. (1999). Frequency of alpha- and beta-haemolysin in *Staphylococcus aureus* of bovine and human origin. A comparison between pheno- and genotype and variation in phenotypic expression. *APMIS* 107 425–430 10230698

[B2] Abdelzaher A. M. Wright M. E. Ortega C. Solo-Gabriele H. M. Miller G. Elmir S. Newman X. Shih P. Bonilla J. A. Bonilla T. D. Palmer C. J. Scott T. Lukasik J. Harwood V. J. Mcquaig S. Sinigalliano C, Gidley, M. Plano L. R. Zhu X. Wang J. D. Fleming L. E. (2010). Presence of pathogens and indicator microbes at a non-point source subtropical recreational marine beach. *Appl. Environ. Microbiol.* 76 724–732 19966020 10.1128/AEM.02127-09 PMC2812993

[B3] Agerer F. Lux S. Michel A. Rohde M. Ohlsen K. Hauck C. R. (2005). Cellular invasion by *Staphylococcus aureus* reveals a functional link between focal adhesion kinase and cortactin in integrin-mediated internalisation. *J. Cell Sci.* 118 2189–2200 15855238 10.1242/jcs.02328

[B4] Agerer F. Michel A. Ohlsen K. Hauck C. R. (2003). Integrin-mediated invasion of *Staphylococcus aureus* into human cells requires Src family protein-tyrosine kinases. *J. Biol. Chem.* 278 42524–42531 12893831 10.1074/jbc.M302096200

[B5] Ahmed S. Meghji S. Williams R. J. Henderson B. Brock J. H. Nair S. P. (2001). *Staphylococcus aureus* fibronectin binding proteins are essential for internalization by osteoblasts but do not account for differences in intracellular levels of bacteria. *Infect Immun.* 69 2872–2877 11292701 10.1128/IAI.69.5.2872-2877.2001 PMC98237

[B6] Balaban N. Novick R. P. (1995). Translation of RNAIII, the *Staphylococcus aureus agr* regulatory RNA molecule, can be activated by a 3′-end deletion. *FEMS Microbiol Lett.* 133 155–161 8566701 10.1111/j.1574-6968.1995.tb07877.x

[B7] Bantel H. Sinha B. Domschke W. Peters G. Schulze-Osthoff K. Janicke R. U. (2001). alpha-Toxin is a mediator of *Staphylococcus aureus-induced* cell death and activates caspases via the intrinsic death pathway independently of death receptor signaling. *J. Cell Biol.* 155 637–648 11696559 10.1083/jcb.200105081 PMC2198876

[B8] Bayles K. W. Wesson C. A. Liou L. E. Fox L. K. Bohach G. A. Trumble W. R. (1998). Intracellular *Staphylococcus aureus* escapes the endosome and induces apoptosis in epithelial cells. *Infect. Immun.* 66 336–342 9423876 10.1128/iai.66.1.336-342.1998 PMC107895

[B9] Bergsbaken T. Fink S. L. Cookson B. T. (2009). Pyroptosis: host cell death and inflammation. *Nat. Rev. Microbiol.* 7 99–109 19148178 10.1038/nrmicro2070 PMC2910423

[B10] Bhakdi S. Bayley H. Valeva A. Walev I. Walker B. Kehoe M. Palmer M. (1996). Staphylococcal alpha-toxin, streptolysin-O, and *Escherichia coli* hemolysin: prototypes of pore-forming bacterial cytolysins. *Arch. Microbiol.* 165 73–79 8593102 10.1007/s002030050300

[B11] Bhakdi S. Tranum-Jensen J. (1991). Alpha-toxin of *Staphylococcus aureus*. *Microbiol. Rev.* 55 733–751 1779933 10.1128/mr.55.4.733-751.1991 PMC372845

[B12] Bingham R. J. Rudino-Pinera E. Meenan N. A. Schwarz-Linek U. Turkenburg J. P. Hook M. Garman E. F. Potts J. R. (2008). Crystal structures of fibronectin-binding sites from *Staphylococcus aureus* FnBPA in complex with fibronectin domains. *Proc. Natl. Acad. Sci. U.S.A.* 105 12254–12258 18713862 10.1073/pnas.0803556105 PMC2518095

[B13] Birmingham C. L. Canadien V. Kaniuk N. A. Steinberg B. E. Higgins D. E. Brumell J. H. (2008). Listeriolysin O allows *Listeria monocytogenes* replication in macrophage vacuoles. *Nature* 451 350–354 18202661 10.1038/nature06479

[B14] Brady N. R. Hamacher-Brady A. Yuan H. Gottlieb R. A. (2007). The autophagic response to nutrient deprivation in the hl-1 cardiac myocyte is modulated by Bcl-2 and sarco/endoplasmic reticulum calcium stores. *FEBS J.* 274 3184–3197 17540004 10.1111/j.1742-4658.2007.05849.x

[B15] Buisman H. P. Buys L. F. Langermans J. A. Van Den Broek P. J. Van Furth R. (1991). Effect of probenecid on phagocytosis and intracellular killing of *Staphylococcus aureus* and *Escherichia coli* by human monocytes and granulocytes. *Immunology* 74 338–341 1748482 PMC1384615

[B16] Bulger R. J. Bulger R. E. (1967). Ultra structure of small colony variants of a methicillin-resistant *Staphylococcus aureus*. *J. Bacteriol.* 94 1244–1246 5183077 10.1128/jb.94.4.1244-1246.1967 PMC276799

[B17] Campoy E. Colombo M. I. (2009). Autophagy in intracellular bacterial infection. *Biochim. Biophys. Acta* 1793 1465–1477 19303905 10.1016/j.bbamcr.2009.03.003

[B18] Carnes E. C. Lopez D. M. Donegan N. P. Cheung A. Gresham H. Timmins G. S. Brinker C. J. (2009). Confinement-induced quorum sensing of individual *Staphylococcus aureus* bacteria. *Nat. Chem. Biol.* 6 41–45 19935660 10.1038/nchembio.264 PMC4201857

[B19] Chatterjee I. Kriegeskorte A. Fischer A. Deiwick S. Theimann N. Proctor R. A. Peters G. Herrmann M. Kahl B. C. (2008). In vivo mutations of thymidylate synthase (encoded by *thy*A) are responsible for thymidine dependency in clinical small-colony variants of *Staphylococcus aureus*. *J. Bacteriol.* 190 834–842 17905979 10.1128/JB.00912-07 PMC2223566

[B20] Claro T. Widaa A. O’Seaghdha M. Miajlovic H. Foster T. J. O’Brien F. J. Kerrigan S. W. (2011). *Staphylococcus aureus* protein A binds to osteoblasts and triggers signals that weaken bone in osteomyelitis. *PLoS ONE* 6 e18748 10.1371/journal. pone.0018748 PMC3078117 21525984

[B21] Craven R. R. Gao X. Allen I. C. Gris D. Bubeck Wardenburg J. Mcelvania-Tekippe E. Ting J. P. Duncan J. A. (2009). *Staphylococcus aureus* α-hemolysin activates the NLRP3-inflammasome in human and mouse monocytic cells. *PLoS ONE* 4 e7446 10.1371/journal. pone.0007446 PMC2758589 19826485

[B22] Diep B. A. Gill S. R. Chang R. F. Phan T. H. Chen J. H. Davidson M. G. Lin F. Lin J. Carleton H. A. Mongodin E. F. Sensabaugh G. F. Perdreau-Remington F. (2006). Complete genome sequence of USA300, an epidemic clone of community-acquired meticillin-resistant *Staphylococcus aureus*. *Lancet* 367 731–739 16517273 10.1016/S0140-6736(06)68231-7

[B23] Dorn B. R. Dunn W. A. Jr. Progulske-Fox A. (2002). Bacterial interactions with the autophagic pathway. *Cell. Microbiol.* 4 1–10 11856168 10.1046/j.1462-5822.2002.00164.x

[B24] Dramsi S. Cossart P. (2002). Listeriolysin O: a genuine cytolysin optimized for an intracellular parasite. *J. Cell Biol.* 156 943–946 11901162 10.1083/jcb.200202121 PMC2173465

[B25] Dziewanowska K. Carson A. R. Patti J. M. Deobald C. F. Bayles K. W. Bohach G. A. (2000). Staphylococcal fibronectin binding protein interacts with heat shock protein 60 and integrins: role in internalization by epithelial cells. *Infect. Immun.* 68 6321–6328 11035741 10.1128/iai.68.11.6321-6328.2000 PMC97715

[B26] Dziewanowska K. Patti J. M. Deobald C. F. Bayles K. W. Trumble W. R. Bohach G. A. (1999). Fibronectin binding protein and host cell tyrosine kinase are required for internalization of *Staphylococcus aureus* by epithelial cells. *Infect. Immun.* 67 4673–4678 10456915 10.1128/iai.67.9.4673-4678.1999 PMC96793

[B27] Edwards A. M. Potter U. Meenan N. A. Potts J. R. Massey R. C. (2011). *Staphylococcus aureus* keratinocyte invasionisdependent upon multiple high-affinity fibronectin-binding repeats within FnBPA. *PLoS ONE* 6 e18899 10.1371/journal. pone.0018899 PMC3081306 21526122

[B28] Edwards A. M. Potts J. R. Josefsson E. Massey R. C. (2010). *Staphylococcus aureus* host cell invasion and virulence in sepsis is facilitated by the multiple repeats within FnBPA. *PLoS Pathog.* 6 e1000964 10.1371/journal.ppat.1000964 PMC2891841 20585570

[B29] Ellington J. K. Elhofy A. Bost K. L. Hudson M. C. (2001). Involvement of mitogen-activated protein kinase pathways in *Staphylococcus aureus* invasion of normal osteoblasts. *Infect. Immun.* 69 5235–5242 11500391 10.1128/IAI.69.9.5235-5242.2001 PMC98631

[B30] Essmann F. Bantel H. Totzke G. Engels I. H. Sinha B. Schulze-Osthoff K. Janicke R. U. (2003). *Staphylococcus aureus* alpha-toxin-induced cell death: predominant necrosis despite apoptotic caspase activation. *Cell Death Differ.* 10 1260–1272 12894214 10.1038/sj.cdd.4401301

[B31] Fowler T. Wann E. R. Joh D. Johansson S. Foster T. J. Hook M. (2000). Cellular invasion by *Staphylococcus aureus* involves a fibronectin bridge between the bacterial fibronectin-binding MSCRAMMs and host cell beta1 integrins. *Eur. J. Cell Biol.* 79 672–679 11089915 10.1078/0171-9335-00104

[B32] Garzoni C. Francois P. Huyghe A. Couzinet S. Tapparel C. Charbonnier Y. Renzoni A. Lucchini S. Lew D. P. Vaudaux P. Kelley W. L. Schrenzel J. (2007). A global view of *Staphylococcus aureus* whole genome expression upon internalization in human epithelial cells. *BMC Genomics* 8 171 10.1186/ 1471-2164-8-171 PMC1924023 17570841

[B33] Garzoni C. Kelley W. L. (2009). *Staphylococcus aureus*: new evidence for intracellular persistence. *Trends Microbiol.* 17 59–65 19208480 10.1016/j.tim.2008.11.005

[B34] Gawronski J. D. Wong S. M. Giannoukos G. Ward D. V. Akerley B. J. (2009). Tracking insertion mutants within libraries by deep sequencing and a genome-wide screen for *Haemophilus* genes required in the lung. *Proc. Natl. Acad. Sci. U.S.A.* 106 16422–16427 19805314 10.1073/pnas.0906627106 PMC2752563

[B35] Genestier A. L. Michallet M. C. Prévost G. Bellot G. Chalabreysse L. Peyrol S. Thivolet F. Etienne J. Lina G. Vallette F. M. Vandenesch F. Genestier L. (2005). *Staphylococcus aureus* Panton-Valentine leukocidin directly targets mitochondria and induces Bax-independent apoptosis of human neutrophils. *J. Clin. Invest.* 115 3117–3127 16276417 10.1172/JCI22684 PMC1265849

[B36] Giese B. Dittmann S. Paprotka K. Levin K. Weltrowski A. Biehler D. Lam T. T. Sinha B. Fraunholz M. J. (2009). Staphylococcal alpha-toxin is not sufficient to mediate escape from phagolysosomes in upper-airway epithelial cells. *Infect. Immun.* 77 3611–3625 19564384 10.1128/IAI.01478-08 PMC2738027

[B37] Giese B. Glowinski F. Paprotka K. Dittmann S. Steiner T. Sinha B. Fraunholz M. J. (2011). Expression of delta-toxin by *Staphylococcus aureus* mediates escape from phago-endosomes of human epithelial and endothelial cells in the presence of beta-toxin. *Cell. Microbiol.* 13 316–329 20946243 10.1111/j.1462-5822.2010.01538.x

[B38] Goerke C. Pantucek R. Holtfreter S. Schulte B. Zink M. Grumann D. Broker B. M. Doskar J. Wolz C. (2009). Diversity of prophages in dominant *Staphylococcus aureus* clonal lineages. *J. Bacteriol.* 191 3462–3468 19329640 10.1128/JB.01804-08 PMC2681900

[B39] Gomez M. I. Prince A. (2008). Airway epithelial cell signaling in response to bacterial pathogens. *Pediatr. Pulmonol.* 43 11–19 18041080 10.1002/ppul.20735

[B40] Gresham H. D. Lowrance J. H. Caver T. E. Wilson B. S. Cheung A. L. Lindberg F. P. (2000). Survival of *Staphylococcus aureus* inside neutrophils contributes to infection 1. *J. Immunol.* 164 3713–3722 10725730 10.4049/jimmunol.164.7.3713

[B41] Haim M. Trost A. Maier C. J. Achatz G. Feichtner S. Hintner H. Bauer J. W. Onder K. (2010). Cytokeratin 8 interacts with clumping factor B: a new possible virulence factor target. *Microbiology* 156 3710–3721 20817646 10.1099/mic.0.034413-0

[B42] Hakansson A. Bentley C. C. Shakhnovic E. A. Wessels M. R. (2005). Cytolysin-dependent evasion of lysosomal killing. *Proc. Natl. Acad. Sci. U.S.A.* 102 5192–5197 15795386 10.1073/pnas.0408721102 PMC555683

[B43] Hamill R. J. Vann J. M. Proctor R. A. (1986). Phagocytosis of *Staphylococcus aureus* by cultured bovine aortic endothelial cells: model for postadherence events in endovascular infections. *Infect. Immun.* 54 833–836 3781627 10.1128/iai.54.3.833-836.1986 PMC260245

[B44] Harraghy N. Hussain M. Haggar A. Chavakis T. Sinha B. Herrmann M. Flock J. I. (2003). The adhesive and immunomodulating properties of the multifunctional *Staphylococcus aureus* protein Eap. *Microbiology* 149 2701–2707 14523103 10.1099/mic.0.26465-0

[B45] Haslinger-Löffler B. Kahl B. C. Grundmeier M. Strangfeld K. Wagner B. Fischer U. Cheung A. L. Peters G. Schulze-Osthoff K. Sinha B. (2005). Multiple virulence factors are required for *Staphylococcus aureus*-induced apoptosis in endothelial cells. *Cell. Microbiol.* 7 1087–1097 16008576 10.1111/j.1462-5822.2005.00533.x

[B46] Haslinger B. Strangfeld K. Peters G. Schulze-Osthoff K. Sinha B. (2003). *Staphylococcus aureus* alpha-toxin induces apoptosis in peripheral blood mononuclear cells: role of endogenous tumour necrosis factor-alpha and the mitochondrial death pathway. *Cell. Microbiol.* 5 729–741 12969378 10.1046/j.1462-5822.2003.00317.x

[B47] Hiemstra P. S. Annema A. Schippers E. F. Van Furth R. (1992). Pertussis toxin partially inhibits phagocytosis of immunoglobulin G-opsonized *Staphylococcus aureus* by human granulocytes but does not affect intracellular killing. *Infect. Immun.* 60 202–205 1309512 10.1128/iai.60.1.202-205.1992 PMC257523

[B48] Hirschhausen N. Schlesier T. Schmidt M. A. Gotz F. Peters G. Heilmann C. (2010). A novel staphylococcal internalization mechanism involves the major autolysin Atl and heat shock cognate protein Hsc70 as host cell receptor. *Cell. Microbiol.* 12 1746–1764 20642807 10.1111/j.1462-5822.2010.01506.x

[B49] Horsburgh M. J. Aish J. L. White I. J. Shaw L. Lithgow J. K. Foster S. J. (2002). sigmaB modulates virulence determinant expression and stress resistance: characterization of a functional rsbU strain derived from *Staphylococcus aureus* 8325-4. *J. Bacteriol.* 184 5457–5467 12218034 10.1128/JB.184.19.5457-5467.2002 PMC135357

[B50] Hruz P. Zinkernagel A. S. Jenikova G. Botwin G. J. Hugot J. P. Karin M. Nizet V. Eckmann L. (2009). NOD2 contributes to cutaneous defense against *Staphylococcus aureus* through alpha-toxin-dependent innate immune activation. *Proc. Natl. Acad. Sci. U.S.A.* 106 12873–12878 19541630 10.1073/pnas.0904958106 PMC2722361

[B51] Husmann M. Beckmann E. Boller K. Kloft N. Tenzer S. Bobkiewicz W. Neukirch C. Bayley H. Bhakdi S. (2009). Elimination of a bacterial pore-forming toxin by sequential endocytosis and exocytosis. *FEBS Lett.* 583 337–344 19101547 10.1016/j.febslet.2008.12.028

[B52] Inoshima I. Inoshima N. Wilke G. A. Powers M. E. Frank K. M. Wang Y. Bubeck Wardenburg J. (2011). A *Staphylococcus aureus* pore-forming toxin subverts the activity of ADAM10 to cause lethal infection in mice. *Nat. Med.* 17 1310–1314 21926978 10.1038/nm.2451 PMC3192248

[B53] Imre G. Heering J. Takeda A. N. Husmann M. Thiede B. zu Heringdorf D. M. Green D. R. der Goot F. G. Sinha B. Dötsch V. Rajalingam K. (2012). Caspase-2 is an initiator caspase responsible for pore-forming toxin-mediated apoptosis. *EMBO J.* (in press) 10.1038/emboj.2012.93 PMC3365430 22531785

[B54] Jarry T. M. Cheung A. L. (2006). *Staphylococcus aureus* escapes more efficiently from the phagosome of a cystic fibrosis bronchial epithelial cell line than from its normal counterpart. *Infect. Immun.* 74 2568–2577 16622192 10.1128/IAI.74.5.2568-2577.2006 PMC1459703

[B55] Jarry T. M. Memmi G. Cheung A. L. (2008). The expression of alpha-haemolysin is required for *Staphylococcus aureus* phagosomal escape after internalization in CFT-1 cells. *Cell. Microbiol.* 10 1801–1814 18466345 10.1111/j.1462-5822.2008.01166.x

[B56] Jevon M. Guo C. Ma B. Mordan N. Nair S. P. Harris M. Henderson B. Bentley G. Meghji S. (1999). Mechanisms of internalization of *Staphylococcus aureus* by cultured human osteoblasts. *Infect. Immun.* 67 2677–2681 10225942 10.1128/iai.67.5.2677-2681.1999 PMC116025

[B57] Kahl B. C. Goulian M. Van Wamel W. Herrmann M. Simon S. M. Kaplan G. Peters G. Cheung A. L. (2000). *Staphylococcus aureus* RN6390 replicates and induces apoptosis in a pulmonary epithelial cell line. *Infect. Immun.* 68 5385–5392 10948168 10.1128/iai.68.9.5385-5392.2000 PMC101802

[B58] Kapetanovic R. Nahori M. A. Balloy V. Fitting C. Philpott D. J. Cavaillon J. M. Adib-Conquy M. (2007). Contribution of phagocytosis and intracellular sensing for cytokine production by *Staphylococcus aureus*-activated macrophages. *Infect. Immun.* 75 830–837 17118979 10.1128/IAI.01199-06 PMC1828506

[B59] Kapral F. A. (1976). Effect of fatty acids on *Staphylococcus aureus* delta-toxin hemolytic activity. *Infect. Immun.* 13 114–119 1248865 10.1128/iai.13.1.114-119.1976 PMC420584

[B60] Kebaier C. Chamberland R. R. Allen I. C. Gao X. Broglie P. M. Hall J. D. Jania C. Doerschuk C. M. Tilley S. L. Duncan J. A. (2012). *Staphylococcus aureus* alpha-hemolysin mediates virulence in a murine model of severe pneumonia through activation of the NLRP3 inflammasome. *J. Infect. Dis.* 205 807–817 22279123 10.1093/infdis/jir846 PMC3274379

[B61] Kerdudou S. Laschke M. W. Sinha B. Preissner K. T. Menger M. D. Herrmann M. (2006). Fibronectin binding proteins contribute to the adherence of *Staphylococcus aureus* to intact endothelium in vivo. *Thromb. Haemost.* 96 183–189 16894462

[B62] Kintarak S. Whawell S. A. Speight P. M. Packer S. Nair S. P. (2004). Internalization of *Staphylococcus aureus* by human keratinocytes. *Infect. Immun.* 72 5668–5675 15385465 10.1128/IAI.72.10.5668-5675.2004 PMC517534

[B63] Kipp F. Ziebuhr W. Becker K. Krimmer V. Hobeta N. Peters G. von Eiff C. (2003). Detection of *Staphylococcus aureus* by 16S rRNA directed in situ hybridisation in a patient with a brain abscess caused by small colony variants. *J. Neurol. Neurosurg. Psychiatry* 74 1000–1002 12810807 10.1136/jnnp.74.7.1000 PMC1738524

[B64] Kirkegaard K. Taylor M. P. Jackson W. T. (2004). Cellular autophagy: surrender, avoidance and subversion by microorganisms. *Nat. Rev. Microbiol.* 2 301–314 15031729 10.1038/nrmicro865 PMC7097095

[B65] Koziel J. Maciag-Gudowska A. Mikolajczyk T. Bzowska M. Sturdevant D. E. Whitney A. R. Shaw L. N. Deleo F. R. Potempa J. (2009). Phagocytosis of *Staphylococcus aureus* by macrophages exerts cytoprotective effects manifested by the upregulation of antiapoptotic factors. *PLoS ONE* 4 e5210 10.1371/journal. pone.0005210 PMC2668171 19381294

[B66] Kreger A. S. Bernheimer A. W. (1971). Disruption of bacterial protoplasts and spheroplasts by staphylococcal delta hemolysin. *Infect. Immun.* 3 603–605 16558024 10.1128/iai.3.4.603-605.1971 PMC416203

[B67] Kreger A. S. Kim K. S. Zaboretzky F. Bernheimer A. W. (1971). Purification and properties of staphylococcal delta hemolysin. *Infect. Immun.* 3 449–465 16557995 10.1128/iai.3.3.449-465.1971 PMC416173

[B68] Kriegeskorte A. Konig S. Sander G. Pirkl A. Mahabir E. Proctor R. A. von Eiff C. Peters G. Becker K. (2011). Small colony variants of *Staphylococcus aureus* reveal distinct protein profiles. *Proteomics* 11 2476–2490 21595038 10.1002/pmic.201000796

[B69] Krut O. Utermohlen O. Schlossherr X. Kronke M. (2003). Strain-specific association of cytotoxic activity and virulence of clinical *Staphylococcus aureus* isolates. *Infect. Immun.* 71 2716–2723 12704146 10.1128/IAI.71.5.2716-2723.2003 PMC153241

[B70] Kubica M. Guzik K. Koziel J. Zarebski M. Richter W. Gajkowska B. Golda A. Maciag-Gudowska A. Brix K. Shaw L. Foster T. Potempa J. (2008). A potential new pathway for *Staphylococcus aureus* dissemination: the silent survival of *S. aureus* phagocytosed by human monocyte-derived macrophages. *PLoS ONE* 3 e1409 10.1371/journal.pone.0001409 PMC2169301 18183290

[B71] Lám T. T. Giese B. Chikkaballi D. Kuhn A. Wolber W. Pané-Farré J. Schäfer D. Engelmann S. Fraunholz M. Sinha B. (2010). Phagolysosomal integrity is generally maintained after *Staphylococcus aureus* invasion of nonprofessional phagocytes but is modulated by strain 6850. *Infect. Immun.* 78 3392–3403 20530231 10.1128/IAI.00012-10 PMC2916288

[B72] Lammers A. Nuijten P. J. M. Smith H. E. (1999). The fibronectin binding proteins of *Staphylococcus aureus* are required for adhesion to and invasion of bovine mammary gland cells. *FEMS Microbiol. Lett.* 180 103–109 10547450 10.1111/j.1574-6968.1999.tb08783.x

[B73] Laschke M. W. Kerdudou S. Herrmann M. Menger M. D. (2005). Intravital fluorescence microscopy: a novel tool for the study of the interaction of *Staphylococcus aureus* with the microvascular endothelium in vivo. *J. Infect. Dis.* 191 435–443 15633103 10.1086/427193

[B74] Levine B. (2005). Eating oneself and uninvited guests: autophagy-related pathways in cellular defense. *Cell* 120 159–162 15680321 10.1016/j.cell.2005.01.005

[B75] Li M. Rigby K. Lai Y. Nair V. Peschel A. Schittek B. Otto M. (2009). *Staphylococcus aureus* mutant screen reveals interaction of the human antimicrobial peptide dermcidin with membrane phospholipids. *Antimicrob. Agents Chemother.* 53 4200–4210 19596877 10.1128/AAC.00428-09 PMC2764178

[B76] Loffler B. Hussain M. Grundmeier M. Bruck M. Holzinger D. Varga G. Roth J. Kahl B. C. Proctor R. A. Peters G. (2010). *Staphylococcus aureus* Panton-Valentine leukocidin is a very potent cytotoxic factor for human neutrophils. *PLoS Pathog.* 6 e1000715 10.1371/journal. ppat.1000715 PMC2798753 20072612

[B77] Lowy F. D. Fant J. Higgins L. L. Ogawa S. K. Hatcher V. B. (1988). *Staphylococcus aureus* – human endothelial cell interactions. *J. Ultrastruct. Mol. Struct. Res.* 98 137–146 3373067 10.1016/s0889-1605(88)80906-6

[B78] Mariathasan S. Weiss D. S. Newton K. Mcbride J. O’Rourke K. Roose-Girma M. Lee W. P. Weinrauch Y. Monack D. M. Dixit V. M. (2006). Cryopyrin activates the inflammasome in response to toxins and ATP. *Nature* 440 228–232 16407890 10.1038/nature04515

[B79] Menzies B. E. Kourteva I. (1998). Internalization of *Staphylococcus aureus* by endothelial cells induces apoptosis. *Infect. Immun.* 66 5994–5998 9826383 10.1128/iai.66.12.5994-5998.1998 PMC108759

[B80] Menzies B. E. Kourteva I. (2000). *Staphylococcus aureus* alpha-toxin induces apoptosis in endothelial cells. *FEMS Immunol. Med. Microbiol.* 29 39–45 10967259 10.1111/j.1574-695X.2000.tb01503.x

[B81] Mestre M. B. Fader C. M. Sola C. Colombo M. I. (2010). Alpha-hemolysin is required for the activation of the autophagic pathway in *Staphylococcus aureus*-infected cells. *Autophagy* 6 110–125 20110774 10.4161/auto.6.1.10698

[B82] Mohammed K. A. Nasreen N. Antony V. B. (2007). Bacterial induction of early response genes and activation of proapoptotic factors in pleural mesothelial cells. *Lung* 185 355–365 17929089 10.1007/s00408-007-9046-6

[B83] Moisan H. Brouillette E. Jacob C. L. Langlois-Begin P. Michaud S. Malouin F. (2006). Transcription of virulence factors in *Staphylococcus aureus* small-colony variants isolated from cystic fibrosis patients is influenced by SigB. *J. Bacteriol.* 188 64–76 16352822 10.1128/JB.188.1.64-76.2006 PMC1317593

[B84] Munoz-Planillo R. Franchi L. Miller L. S. Nunez G. (2009). A critical role for hemolysins and bacterial lipoproteins in *Staphylococcus aureus*-induced activation of the Nlrp3 inflammasome. *J. Immunol.* 183 3942–3948 19717510 10.4049/jimmunol.0900729 PMC2762867

[B85] Nuzzo I. Sanges M. R. Folgore A. Carratelli C. R. (2000). Apoptosis of human keratinocytes after bacterial invasion. *FEMS Immunol. Med. Microbiol.* 27 235–240 10683468 10.1111/j.1574-695X.2000.tb01435.x

[B86] O’Brien L. M. Walsh E. J. Massey R. C. Peacock S. J. Foster T. J. (2002). *Staphylococcus aureus* clumping factor B (ClfB) promotes adherence to human type I cytokeratin 10: implications for nasal colonization. *Cell. Microbiol.* 4 759–770 12427098 10.1046/j.1462-5822.2002.00231.x

[B87] Ogawa M. Mimuro H. Yoshikawa Y. Ashida H. Sasakawa C. (2011). Manipulation of autophagy by bacteria for their own benefit. *Microbiol. Immunol.* 55 459–471 21707736 10.1111/j.1348-0421.2011.00343.x

[B88] Olivier A. C. Lemaire S. Van Bambeke F. Tulkens P. M. Oldfield E. (2009). Role of rsbU and staphyloxanthin in phagocytosis and intracellular growth of *Staphylococcus aureus* in human macrophages and endothelial cells. *J. Infect. Dis.* 200 1367–1370 19817587 10.1086/606012 PMC2762113

[B89] Orvedahl A. Levine B. (2009). Eating the enemy within: autophagy in infectious diseases. *Cell Death Differ.* 16 57–69 18772897 10.1038/cdd.2008.130 PMC2736877

[B90] Otto M. O’Mahoney D. S. Guina T. Klebanoff S. J. (2004). Activity of *Staphylococcus epidermidis* phenol-soluble modulin peptides expressed in *Staphylococcus carnosus*. *J. Infect. Dis.* 190 748–755 15272403 10.1086/422157

[B91] Oviedo-Boyso J. Cortes-Vieyra R. Huante-Mendoza A. Yu H. B. Valdez-Alarcon J. J. Bravo-Patino A. Cajero-Juarez M. Finlay B. B. Baizabal-Aguirre V. M. (2011). The phosphoinositide-3-kinase-Akt signaling pathway is important for *Staphylococcus aureus* internalization by endothelial cells. *Infect. Immun.* 79 4569–4577 21844240 10.1128/IAI.05303-11 PMC3257907

[B92] Pang Y. Y. Schwartz J. Thoendel M. Ackermann L. W. Horswill A. R. Nauseef W. M. (2010). agr-Dependent interactions of *Staphylococcus aureus* USA300 with human polymorphonuclear neutrophils. *J. Innate Immun.* 2 546–559 20829608 10.1159/000319855 PMC2982852

[B93] Peacock S. J. Foster T. J. Cameron B. J. Berendt A. R. (1999). Bacterial fibronectin-binding proteins and endothelial cell surface fibronectin mediate adherence of *Staphylococcus aureus* to resting human endothelial cells. *Microbiology* 145(Pt. 12) 3477–3486 10627045 10.1099/00221287-145-12-3477

[B94] Petrilli V. Papin S. Dostert C. Mayor A. Martinon F. Tschopp J. (2007). Activation of the NALP3 inflammasome is triggered by low intracellular potassium concentration. *Cell Death Differ.* 14 1583–1589 17599094 10.1038/sj.cdd.4402195

[B95] Pokorny A. Yandek L. E. Elegbede A. I. Hinderliter A. Almeida P. F. (2006). Temperature and composition dependence of the interaction of delta-lysin with ternary mixtures of sphingomyelin/cholesterol/POPC. *Biophys. J.* 91 2184–2197 16798807 10.1529/biophysj.106.085027 PMC1557559

[B96] Proctor R. A. Balwit J. M. Vesga O. (1994). Variant subpopulations of *Staphylococcus aureus* as cause of persistent and recurrent infections. *Infect. Agents Dis.* 3 302–312 7889317

[B97] Proctor R. A. Van Langevelde P. Kristjansson M. Maslow J. N. Arbeit R. D. (1995). Persistent and relapsing infections associated with small-colony variants of *Staphylococcus aureus*. *Clin. Infect. Dis.* 20 95–102 7727677 10.1093/clinids/20.1.95

[B98] Qazi S. N. A. Counil E. Morrissey J. Rees C. E. D. Cockayne A. Winzer K. Chan W. C. Williams P. Hill P. J. (2001). *agr* expression precedes escape of internalized *Staphylococcus aureus* from the host endosome. *Infect. Immun.* 69 7074–7082 11598083 10.1128/IAI.69.11.7074-7082.2001 PMC100088

[B99] Rahal J. J. Jr. (1972). Comparative effects of purified staphylococcal alpha and delta toxins on mitochondrial metabolism. *J. Infect. Dis.* 126 96–103 4260986 10.1093/infdis/126.1.96

[B100] Rudel T. Kepp O. Kozjak-Pavlovic V. (2010). Interactions between bacterial pathogens and mitochondrial cell death pathways. *Nat. Rev. Microbiol.* 8 693–705 20818415 10.1038/nrmicro2421

[B101] Rudino-Pinera E. Schwarz-Linek U. Potts J. R. Garman E. F. (2004). Twinned or not twinned, that is the question: crystallization and preliminary crystallographic analysis of the 2F1(3)F1 module pair of human fibronectin. *Acta Crystallogr. D Biol. Crystallogr.* 60 1341–1345 15213410 10.1107/S0907444904011473

[B102] Schnaith A. Kashkar H. Leggio S. A. Addicks K. Krönke M. Krut O. (2007). *Staphylococcus aureus* subvert autophagy for induction of caspase-independent host cell death. *J. Biol. Chem.* 282 2695–2706 17135247 10.1074/jbc.M609784200

[B103] Schnupf P. Portnoy D. A. (2007). Listeriolysin O: a phagosome-specific lysin. *Microbes Infect.* 9 1176–1187 17720603 10.1016/j.micinf.2007.05.005

[B104] Schröder A. Kland R. Peschel A. von Eiff C. Aepfelbacher M. (2006a). Live cell imaging of phagosome maturation in *Staphylococcus aureus* infected human endothelial cells: small colony variants are able to survive in lysosomes. *Med. Microbiol. Immunol.* 195 185–194 10.1007/s00430-006-0015-0 16596413

[B105] Schröder A. Schröder B. Roppenser B. Linder S. Sinha B. Fassler R. Aepfelbacher M. (2006b). *Staphylococcus aureus* fibronectin binding protein-A induces motile attachment sites and complex actin remodeling in living endothelial cells. *Mol. Biol. Cell* 17 5198–5210 10.1091/mbc.E06-05-0463 PMC1679684 17021255

[B106] Schwartz J. Leidal K. G. Femling J. K. Weiss J. P. Nauseef W. M. (2009). Neutrophil bleaching of GFP-expressing staphylococci: probing the intraphagosomal fate of individual bacteria. *J. Immunol.* 183 2632–2641 19620311 10.4049/jimmunol.0804110 PMC2881311

[B107] Schwarz-Linek U. Werner J. M. Pickford A. R. Gurusiddappa S. Kim J. H. Pilka E. S. Briggs J. A. Gough T. S. Hook M. Campbell I. D. Potts J. R. (2003). Pathogenic bacteria attach to human fibronectin through a tandem beta-zipper. *Nature* 423 177–181 12736686 10.1038/nature01589

[B108] Selva L. Viana D. Regev-Yochay G. Trzcinski K. Corpa J. M. Lasa I. Novick R. P. Penades J. R. (2009). Killing niche competitors by remote-control bacteriophage induction. *Proc. Natl. Acad. Sci. U.S.A.* 106 1234–1238 19141630 10.1073/pnas.0809600106 PMC2633583

[B109] Sendi P. Proctor R. A. (2009). *Staphylococcus aureus* as an intracellular pathogen: the role of small colony variants. *Trends Microbiol.* 17 54–58 19162480 10.1016/j.tim.2008.11.004

[B110] Shompole S. Henon K. T. Liou L. E. Dziewanowska K. Bohach G. A. Bayles K. W. (2003). Biphasic intracellular expression of *Staphylococcus aureus* virulence factors and evidence for Agr-mediated diffusion sensing. *Mol. Microbiol.* 49 919–927 12890018 10.1046/j.1365-2958.2003.03618.x

[B111] Sinha B. Francois P. Que Y. A. Hussain M. Heilmann C. Moreillon P. Lew D. Krause K. H. Peters G. Herrmann M. (2000). Heterologously expressed *Staphylococcus aureus* fibronectin-binding proteins are sufficient for invasion of host cells. *Infect. Immun.* 68 6871–6878 11083807 10.1128/iai.68.12.6871-6878.2000 PMC97792

[B112] Sinha B. François P. P. Nüße O. Foti M. Hartford O. M. Vaudaux P. Foster T. J. Lew D. P. Herrmann M. Krause K.-H. (1999). Fibronectin-binding protein acts as *Staphylococcus aureus* invasin via fibronectin bridging to integrin a5β1. *Cell. Microbiol.* 1 101–117 11207545 10.1046/j.1462-5822.1999.00011.x

[B113] Sinha B. Fraunholz M. (2010). *Staphylococcus aureus* host cell invasion and post-invasion events. *Int. J. Med. Microbiol.* 300 170–175 19781990 10.1016/j.ijmm.2009.08.019

[B114] Strober W. Murray P. J. Kitani A. Watanabe T. (2006). Signalling pathways and molecular interactions of NOD1 and NOD2. *Nat. Rev. Immunol.* 6 9–20 16493424 10.1038/nri1747

[B115] Taylor R. C. Cullen S. P. Martin S. J. (2008). Apoptosis: controlled demolition at the cellular level. *Nat. Rev. Mol. Cell Biol.* 9 231–241 18073771 10.1038/nrm2312

[B116] Ting J. P. Lovering R. C. Alnemri E. S. Bertin J. Boss J. M. Davis B. K. Flavell R. A. Girardin S. E. Godzik A. Harton J. A. Hoffman H. M. Hugot J. P. Inohara N. Mackenzie A. Maltais L. J. Nunez G. Ogura Y. Otten L. A. Philpott D. Reed J. C. Reith W. Schreiber S. Steimle V. Ward P. A. (2008a). The NLR gene family: a standard nomenclature. *Immunity* 28 285–287 18341998 10.1016/j.immuni.2008.02.005 PMC2630772

[B117] Ting J. P. Willingham S. B. Bergstralh D. T. (2008b). NLRs at the intersection of cell death and immunity. *Nat. Rev. Immunol.* 8 372–379 18362948 10.1038/nri2296

[B118] Tuchscherr L. Medina E. Hussain M. Volker W. Heitmann V. Niemann S. Holzinger D. Roth J. Proctor R. A. Becker K. Peters G. Loffler B. (2011). *Staphylococcus aureus* phenotype switching: an effective bacterial strategy to escape host immune response and establish a chronic infection. *EMBO Mol. Med.* 3 129–141 21268281 10.1002/emmm.201000115 PMC3395110

[B119] Tucker K. A. Reilly S. S. Leslie C. S. Hudson M. C. (2000). Intracellular *Staphylococcus aureus* induces apoptosis in mouse osteoblasts. *FEMS Microbiol. Lett.* 186 151–156 10802163 10.1111/j.1574-6968.2000.tb09096.x

[B120] Vann J. M. Proctor R. A. (1988). Cytotoxic effects of ingested *Staphylococcus aureus* on bovine endothelial cells: role of *S. aureus* a-hemolysin. *Microb. Pathog.* 4 443–453 3193875 10.1016/0882-4010(88)90029-0

[B121] van Opijnen T. Bodi K. L. Camilli A. (2009). Tn-seq: high-throughput parallel sequencing for fitness and genetic interaction studies in microorganisms. *Nat. Methods* 6 767–772 19767758 10.1038/nmeth.1377 PMC2957483

[B122] Verdon J. Girardin N. Lacombe C. Berjeaud J. M. Hechard Y. (2009). delta-hemolysin, an update on a membrane-interacting peptide. *Peptides* 30 817–823 19150639 10.1016/j.peptides.2008.12.017

[B123] von Eiff C. Peters G. Proctor R. A. (2001). “Small colony variants of *Staphylococcus aureus*: mechanisms for production, biology of infection, and clinical significance,” in *Staphylococcus aureus Infection and Disease*, eds Honeyman A. L. Friedman H. Bendinelli M. (New York, NY: Kluwer Academic/Plenum),17–33

[B124] Wadstrom T. Mollby R. (1972). Some biological properties of purified staphylococcal haemolysins. *Tox -icon* 10 511–519 10.1016/0041-0101(72)90177-8 5071600

[B125] Walev I. Martin E. Jonas D. Mohamadzadeh M. Muller-Klieser W. Kunz L. Bhakdi S. (1993). Staphylococcal alpha-toxin kills human keratinocytes by permeabilizing the plasma membrane for monovalent ions. *Infect. Immun.* 61 4972–4979 8225571 10.1128/iai.61.12.4972-4979.1993 PMC281271

[B126] Wang R. Braughton K. R. Kretschmer D. Bach T. H. Queck S. Y. Li M. Kennedy A. D. Dorward D. W. Klebanoff S. J. Peschel A. Deleo F. R. Otto M. (2007). Identification of novel cytolytic peptides as key virulence determinants for community-associated MRSA. *Nat. Med.* 13 1510–1514 17994102 10.1038/nm1656

[B127] Weidenmaier C. Kokai-Kun J. F. Kristian S. A. Chanturiya T. Kalbacher H. Gross M. Nicholson G. Neumeister B. Mond J. J. Peschel A. (2004). Role of teichoic acids in *Staphylococcus aureus* nasal colonization, a major risk factor in nosocomial infections. *Nat. Med.* 10 243–245 14758355 10.1038/nm991

[B128] Weidenmaier C. Kokai-Kun J. F. Kulauzovic E. Kohler T. Thumm G. Stoll H. Gotz F. Peschel A. (2008). Differential roles of sortase-anchored surface proteins and wall teichoic acid in *Staphylococcus aureus* nasal colonization. *Int. J. Med. Microbiol.* 298 505–513 18221914 10.1016/j.ijmm.2007.11.006

[B129] Weidenmaier C. Peschel A. Xiong Y. Q. Kristian S. A. Dietz K. Yeaman M. R. Bayer A. S. (2005). Lack of wall teichoic acids in *Staphylococcus aureus* leads to reduced interactions with endothelial cells and to attenuated virulence in a rabbit model of endocarditis. *J. Infect. Dis.* 191 1771–1777 15838806 10.1086/429692

[B130] Wertheim H. F. Walsh E. Choudhurry R. Melles D. C. Boelens H. A. Miajlovic H. Verbrugh H. A. Foster T. Van Belkum A. (2008). Key role for clumping factor B in *Staphylococcus aureus* nasal colonization of humans. *PLoS Med.* 5 e17 10.1371/journal.pmed. 0050017 PMC2194749 18198942

[B131] Wesson C. A. Deringer J. Liou L. E. Bayles K. W. Bohach G. A. Trumble W. R. (2000). Apoptosis induced by *Staphylococcus aureus* in epithelial cells utilizes a mechanism involving caspases 8 and 3. *Infect. Immun.* 68 2998–3001 10769002 10.1128/iai.68.5.2998-3001.2000 PMC97517

[B132] Wesson C. A. Liou L. E. Todd K. M. Bohach G. A. Trumble W. R. Bayles K. W. (1998). *Staphylococcus aureus* Agr and Sar global regulators influence internalization and induction of apoptosis. *Infect. Immun.* 66 5238–5243 9784528 10.1128/iai.66.11.5238-5243.1998 PMC108654

[B133] Wilke G. A. Bubeck Wardenburg J. (2010). Role of a disintegrin and metalloprotease 10 in *Staphylococcus aureus* alpha-hemolysin-mediated cellular injury. *Proc. Natl. Acad. Sci. U.S.A.* 107 13473–13478 20624979 10.1073/pnas.1001815107 PMC2922128

[B134] Willingham S. B. Ting J. P. (2008). NLRs and the dangers of pollution and aging. *Nat. Immunol.* 9 831–833 18645588 10.1038/ni0808-831 PMC2743174

[B135] Wright J. A. Nair S. P. (2010). Interaction of staphylococci with bone. *Int. J. Med. Microbiol.* 300 193–204 19889575 10.1016/j.ijmm.2009.10.003 PMC2814006

